# Impact of maternal nutrition and gestational weight gain on perinatal outcomes in intrahepatic cholestasis of pregnancy

**DOI:** 10.1590/1806-9282.20251795

**Published:** 2026-06-15

**Authors:** Esra Keles, Leyla Kaya, Yasemin Tugba Ogunc, Zahide Kaya, Kurşad Nuri Baydili, Pınar Kumru

**Affiliations:** 1 University of Health Sciences, Kartal Dr. Lütfi Kırdar City Hospital, Department of Gynecologic Oncology – Istanbul, Turkey.; 2 University of Health Sciences, Faculty of Health Sciences, Department of Midwifery – Istanbul, Turkey.; 3 University of Health Sciences, Hamidiye Faculty of Health Sciences, Department of Nutrition and Dietetics – Istanbul, Turkey.; 4 Uskudar State Hospital, Internal Medicine Clinic – Istanbul, Turkey.; 5 University of Health Sciences Turkey, Hamidiye Faculty of Medicine, Department of Biostatistics – Istanbul, Turkey.; 6 University of Health Sciences, Zeynep Kamil Women and Children's Disease Training and Research Hospital, Department of Obstetrics and Gynecology – Istanbul, Turkey.

**Keywords:** Cholestasis, intrahepatic, Nutritional status, Premature birth, Birth weight, Weight gain, Diet

## Abstract

**OBJECTIVE:**

The aim of this study was to investigate the relationship between intrahepatic cholestasis of pregnancy and maternal nutritional intake, and to evaluate the impact of maternal nutritional status on maternal and fetal outcomes.

**METHODS:**

This prospective case-control study enrolled 38 women with intrahepatic cholestasis of pregnancy and 76 age- and body mass index-matched controls between 2022 and 2023. Dietary intake was assessed using 24-h recall interviews and a 60-item food frequency questionnaire. Nutrient adequacy was calculated relative to age- and pregnancy-specific Dietary Reference Intakes. Maternal anthropometrics and gestational weight gain were recorded, alongside maternal and neonatal outcomes.

**RESULTS:**

Women with intrahepatic cholestasis of pregnancy had significantly lower energy intake (p=0.004) and gestational weight gain (p<0.001). Nutrient adequacy analyses indicated comprehensive deficiencies, with significantly lower adequacy for energy, protein, fiber, folate, magnesium, potassium, and iron (all p<0.01). Intrahepatic cholestasis of pregnancy was associated with earlier delivery (p<0.001), lower neonatal birth weight (p<0.001), and higher neonatal intensive care unit admission rates (p=0.007). Within the intrahepatic cholestasis of pregnancy group, maternal fasting bile acid levels inversely correlated with gestational age at delivery (ρ=-0.408, p=0.011). Gestational weight gain positively correlated with neonatal birth weight (ρ=0.325, p=0.046).

**CONCLUSION:**

Intrahepatic cholestasis of pregnancy is associated with significant maternal nutritional inadequacy and insufficient gestational weight gain. Optimizing maternal nutritional status represents a potentially modifiable pathway for targeted intervention in the comprehensive management of intrahepatic cholestasis of pregnancy.

## INTRODUCTION

Intrahepatic cholestasis of pregnancy (ICP) is recognized as the most frequent reversible liver disorder specific to gestation, affecting approximately 0.1–2% of pregnant women^1^. Clinically, the condition is defined by maternal pruritus and pathological elevation of serum bile acid (BA) concentrations, typically resolving rapidly following delivery. While the maternal prognosis is generally favorable, ICP presents considerable risk to fetal well-being.

The etiology of ICP involves complex interplay among genetic susceptibility, hormonal factors, and environmental influences^2^. Pathophysiologically, impaired hepatocellular secretion of BAs leads to their accumulation in the maternal circulation. These elevated BA concentrations are strongly associated with increased risk of adverse perinatal outcomes, including spontaneous preterm birth, meconium staining, and sudden intrauterine death, especially when BA levels exceed 100 μmol/L^3^. Furthermore, neonates born to mothers with ICP frequently exhibit indicators of fetal growth restriction^4^.

Cholestasis imposes a dual challenge regarding maternal nutritional status. First, impaired bile flow directly impedes the formation of micelles in the intestine. This leads to malabsorption of dietary lipids and fat-soluble vitamins^5^. Second, clinical symptoms such as severe pruritus and potential nausea may substantially decrease maternal appetite and overall caloric intake^6^. Suboptimal nutrient and energy consumption, combined with malabsorption, can rapidly deplete maternal reserves, potentially contributing to inadequate gestational weight gain (GWG)^7^. Although optimizing diet is widely accepted as beneficial for overall pregnancy health, current guidelines often lack specific dietary recommendations for women with ICP due to limited evidence.

The primary objective of this study was to compare the energy and nutrient intake, dietary adequacy relative to established Dietary Reference Intakes (DRIs), and anthropometric indices, including GWG, between pregnant women diagnosed with ICP and healthy controls. The secondary objective was to evaluate the association between maternal nutritional parameters and adverse maternal and neonatal outcomes.

## METHODS

### Study design and population

This prospective case-control study was conducted in the Department of Obstetrics and Gynecology at a tertiary referral hospital between June 2023 and June 2024. The cohort comprised 114 pregnant women: 38 diagnosed with ICP (cases) and 76 age- and body mass index-matched healthy pregnant women (controls). Ethical approval was granted by the Hospital's Ethics Committee (Approval 88, May 24, 2023), following the Declaration of Helsinki tenets. Diagnosis of ICP was based on clinical presentation of pruritus and elevated serum BA concentrations (>10 μmol/L). Patients with other underlying hepatic disorders or systemic diseases that could mimic cholestasis were excluded.

### Data collection and anthropometric measurements

Maternal sociodemographic factors, lifestyle habits, and obstetric history were collected using a standardized questionnaire. All anthropometric assessments were conducted by a dietitian following standardized techniques. Weight and height were recorded to the nearest 0.1 kg and 1 cm, respectively. Body mass index was derived and classified per World Health Organization guidelines^8^. GWG was calculated as the difference between self-reported pre-pregnancy weight and weight measured at delivery. Waist circumference was assessed at the mid-abdominal level during mid-exhalation. Hip circumference was measured at the maximal posterior protrusion of the buttocks.

### Nutritional assessment

Dietary intake assessment was conducted by registered dietitians using validated self-report methods. A combination of 24-h dietary recall and a food frequency questionnaire was employed. The 24-h dietary recall was conducted through a face-to-face method with a Food and Dish Photograph Catalog serving as a retrospective reminder technique^9^. The Food Frequency Questionnaire comprised 60 items, categorized into food groups. For each item, participants reported their consumption frequency over the preceding 3-month period and typical portion size. Daily nutrient intakes were calculated using the Turkish food composition database (BEBIS, Version 8.0)^10^. Nutrient intakes were compared against DRIs established for the Turkish population, adjusted for pregnancy requirements^11^.

### Dietary diversity assessment

Dietary diversity was assessed using a 24-h recall and quantified via the Women's Dietary Diversity Score based on Food and Agriculture Organization guidelines^12^. Dietary intake was categorized into nine food groups. A score of one point was assigned for each food group if the consumed amount was ≥15 g.

### Maternal and neonatal outcomes

Maternal outcomes included gestational age at delivery, mode of delivery, maternal length of hospital stay, and fasting serum BA concentrations. Neonatal outcomes included birth weight, length, head circumference, Apgar scores at 1 and 5 min, need for neonatal intensive care unit admission, and requirement for respiratory support.

### Statistical analysis

Statistical analyses were performed using Statistical Package for the Social Sciences version 28.0. Normality of continuous variables was assessed using the Kolmogorov-Smirnov test. Continuous variables were summarized as mean±standard deviation for normally distributed data or median (minimum–maximum) for skewed data. Categorical variables were expressed as frequencies and percentages. Group comparisons were conducted using Student's t-test or the Mann-Whitney U test for continuous variables, and the chi-square or Fisher's exact test for categorical variables. Correlations were examined using Spearman's rho (ρ). Statistical significance was set at p<0.05.

## RESULTS

### Study population characteristics

The study included 114 pregnant women, with 38 in the ICP group and 76 in the control group. No significant differences were observed between groups regarding maternal education, occupation, family structure, pregnancy planning status, smoking habits, or consumption of coffee and tea. A history of cholestasis in a previous pregnancy was significantly more common in the ICP group (p<0.001). Furthermore, the ICP group exhibited a higher rate of co-existing gestational diabetes compared to controls (47.4 vs. 15.8%; p<0.001).

### Maternal characteristics and anthropometric measurements

Maternal age did not differ significantly between groups (ICP: 30 [22–41] years; Control: 28 [18–38] years; p=0.068). Pre-pregnancy body mass index (25.8±5.6 vs. 25.3±4.7 kg/m²; p=0.759) was comparable between groups. GWG was significantly lower in women with ICP compared to controls (9.3±6.5 vs. 12.9±6.6 kg, respectively; p<0.001) (Table 1).

**Table 1:** Maternal and neonatal characteristics of control and cholestasis groups.

Variable	Control (n=76)	Cholestasis (n=38)	p value
Maternal age (years)	28 (18–38)	30 (22–41)	0.068
Gestational age at delivery (weeks)	39 (35–41.2)	37 (32.3–39.6)	<0.001
Pre-pregnancy BMI (kg/m²)	25.3±4.7	25.8±5.6	0.656
Gestational weight gain (kg)	12.9±6.6	9.3±6.5	<0.001
Waist circumference (cm)	114 (98–130)	109 (92–130)	0.003
Hip circumference (cm)	113 (96–126)	110 (94–132)	0.004
Birth weight (g)	3,272.6±483.0	2,795.5±575.3	<0.001
Birth length (cm)	50.2±2.1	48.3±2.6	<0.001
Head circumference (cm)	35 (31–39)	34 (30–37)	0.009
Apgar score, 1 min	8 (6–8)	8 (4–8)	0.021
Apgar score, 5 min	9 (7–9)	9 (6–9)	0.002
Maternal length of hospital stay (days)	2 (1–5)	10 (3–18)	<0.001
NICU admission, n (%)	11 (27)	14 (56)	0.007
Respiratory support, n (%)	11 (27)	14 (56)	0.007

Values are mean±SD or median (min–max). BMI: body mass index; NICU: neonatal intensive care unit.

### Pregnancy and delivery outcomes

Maternal length of hospital stay was significantly prolonged in the ICP group (median 10 days [range: 3–18] vs. 2 days [range: 1–5], p<0.001). Women with ICP delivered at significantly earlier gestational ages compared to controls (median 37 weeks [range: 32.3–39.6] vs. 39 weeks [range: 35–41.2], p<0.001) (Table 1).

### Neonatal outcomes

Neonatal anthropometric measurements were lower in infants born to mothers with ICP. Birth weight was significantly reduced (2,795.5±575.3 vs. 3,272.6±483.0 g, p<0.001), as were birth length (48.3±2.6 vs. 50.2±2.1 cm, p<0.001) and head circumference (median 34 cm [range: 30–37] vs. 35 cm [range: 31–39], p=0.009). Apgar scores were significantly lower in the ICP group at both 1 min (median 8 [range: 4–8] vs. 8 [range: 6–8], p=0.021) and 5 min (median 9 [range: 6–9] vs. 9 [range: 7–9], p=0.002). Neonates in the ICP group required intensive care admission more frequently than controls (56 vs. 27%, p=0.007) and had higher rates of respiratory support requirements (56 vs. 27%, p=0.007) (Table 1).

### Nutritional intake assessment

Women with ICP demonstrated significantly lower total energy intake compared to controls (median 1,850.7 kcal/day [range: 1,287.1–2,604.2] vs. 2,055.6 kcal/day [range: 1,026.3–3,758.2], p=0.004). Macronutrient analysis revealed significantly reduced intake of protein (64.4 vs. 70.0 g, p=0.023), carbohydrates (199.1 vs. 217.5 g, p=0.027), and total fat (83.0 vs. 95.1 g, p=0.010) in the ICP group.

Fiber intake was significantly lower in women with ICP, with total fiber intake of 39.1 g compared to 47.5 g in controls (p=0.001). both soluble fiber (6.7 vs. 7.9 g, p=0.007) and insoluble fiber (12.4 vs. 14.9 g, p=0.001) were significantly reduced in the ICP group. multiple micronutrient deficiencies were identified in the ICP group. vitamin A intake was significantly lower (median 1,011.5 vs. 1,327.9 μg, p=0.001), as was folate intake from dietary sources (321.1 vs. 381.1 μg, p<0.001), and vitamin C intake (92.7 vs. 112.9 mg, p=0.001). mineral intake analysis revealed significant deficiencies in potassium (2,468.9 vs. 2,926.3 mg, p=0.002), magnesium (261.5 vs. 305.9 mg, p=0.002), and iron (9.2 vs. 10.7 mg, p=0.002) (Table 2).

**Table 2:** Daily nutrient intakes compared with dietary reference intakes.

Nutrient	Control (n=76)	Cholestasis (n=38)	p value (intake)	p value (%DRI)
Energy (kcal)	2,055.6 (1,026–3,758)	1,850.7 (1,287–2,604)	0.004	0.006
Protein (g)	70.0 (41.8–117.9)	64.4 (44.7–101.5)	0.023	0.011
Carbohydrate (g)	217.5 (63.2–433)	199.1 (93.1–313.3)	0.027	0.027
Fat (g)	95.1 (62.7–187.1)	83.0 (54.5–124.3)	0.010	-
Fiber (g)	47.5 (26.2–95.2)	39.1 (27.1–65.2)	0.001	0.001
Vitamin A (μg)	1,327.9 (502–3,214)	1,011.5 (563–3,507)	0.001	0.001
Folate (μg)*	381.1 (180–725)	321.1 (219–474)	<0.001	<0.001
Vitamin C (mg)	112.9 (41–346)	92.7 (47–163)	0.001	0.001
Potassium (mg)	2,926.3 (1,638–4,512)	2,468.9 (1,722–3,775)	0.002	0.002
Magnesium (mg)	305.9 (190–474)	261.5 (176–382)	0.002	0.002
Iron (mg)	10.7 (6.4–17.4)	9.2 (5.9–14.5)	0.002	0.002

Values are median (min–max). DRI: Dietary Reference Intakes.

* Folate from diet only; supplemental folic acid not included.

### Nutrient adequacy compared to dietary reference intakes

When nutrient intakes were compared to established age- and pregnancy-specific DRIs, women with ICP demonstrated significantly lower adequacy percentages across multiple nutrients. Energy adequacy was 82.0% in the ICP group compared to 91.3% in controls (p=0.006). Protein adequacy was 78.6 vs. 87.9% (p=0.011), and fiber adequacy was significantly reduced at 156.4 vs. 190.1% (p=0.001). Micronutrient adequacy patterns showed concerning deficiencies in the ICP group. Folate adequacy was 53.5% compared to 63.5% in controls (p<0.001), vitamin C adequacy was 88.3 vs. 107.5% (p=0.001), and iron adequacy was 57.2 vs. 66.7% (p=0.002) (Table 2).

### Correlation analysis within the intrahepatic cholestasis of pregnancy group

Analysis of associations within the ICP cohort revealed that higher maternal fasting BA concentrations were negatively correlated with gestational age at delivery (ρ=-0.408, p=0.011). Maternal nutritional and anthropometric parameters demonstrated significant positive correlations with fetal growth markers. Neonatal birth weight was positively correlated with maternal GWG (ρ=0.325, p=0.046), waist circumference (ρ=0.474, p=0.003), and hip circumference (ρ=0.384, p=0.017) (Table 3).

**Table 3:** Correlations between maternal parameters and outcomes in the cholestasis group.

Variable	Gestational age (weeks) ρ (p)	Birth weight (g) ρ (p)
Maternal weight gain (kg)	0.236 (0.155)	0.325 (0.046)
Waist circumference (cm)	0.242 (0.144)	0.474 (0.003)
Hip circumference (cm)	0.195 (0.240)	0.384 (0.017)
Fasting bile acids (μmol/L)	-0.408 (0.011)	-0.230 (0.166)

Spearman's rho correlation coefficients shown. p<0.05 considered significant.

## DISCUSSION

The results of this prospective case-control study demonstrated a significant association between ICP and compromised maternal nutritional status. Women with ICP exhibited significantly reduced GWG and inadequacies across multiple macro- and micronutrient categories. These nutritional deficits appear to contribute to adverse maternal and neonatal outcomes, including insufficient GWG, preterm delivery, low birth weight, and increased neonatal morbidity.

The observed reduction in median energy intake (approximately 200 kcal/day) and the significantly lower GWG in the ICP group represent a clinically meaningful difference that falls short of recommended pregnancy requirements. The mechanism underlying reduced energy intake in ICP may relate to pregnancy-associated nausea, early satiety due to hepatomegaly, or psychological factors related to chronic pruritus and sleep disturbance^13^. Suboptimal GWG is a recognized independent risk factor for fetal growth restriction^14^.

Beyond caloric restriction, the findings of this study revealed a broad pattern of nutritional deficiencies, including protein, polyunsaturated fatty acids, and fat-soluble vitamins. This pattern is consistent with the impaired enterohepatic circulation caused by bile flow obstruction^15^. Bile acids are essential for fat emulsification and absorption. Therefore, their reduced availability leads to steatorrhea and malabsorption of fat-soluble nutrients^16^. Furthermore, the observed deficits in essential water-soluble vitamins and minerals suggest a general reduction in dietary quality and intake.

The negative correlation observed between maternal fasting BA concentrations and gestational age at delivery reinforces the critical role of BA severity in determining the timing of delivery^3^. However, the present study further demonstrates that maternal nutritional status acts as a significant modifier of fetal growth, independent of BA concentration. The reduced neonatal birth weight, length, and head circumference confirm the association between ICP and fetal growth restriction, consistent with findings from previous meta-analyses^4^. Importantly, the positive correlation of neonatal birth weight with maternal GWG and other anthropometric reserves suggests that, even in the presence of pathological BA elevation, greater maternal reserves may provide a crucial protective buffer.

The finding that gestational diabetes was prevalent in the ICP group (47.4 vs. 15.8%) suggests a shared metabolic dysfunction, likely involving dysregulated lipid and glucose homeostasis that is exacerbated by elevated BAs^17^. This comorbidity adds complexity to nutritional management. Dietary optimization must address both caloric adequacy for GWG and carbohydrate control for diabetes management.

The findings of the present study indicate that clinical management protocols for ICP should be broadened beyond current approaches. While existing protocols primarily focus on ursodeoxycholic acid and timely delivery planning, they should also incorporate systematic nutritional screening and targeted intervention. Given the comprehensive inadequacies observed, particularly in nutrients such as folate, iron, and protein, specialized dietary assessment and counseling are recommended for all women diagnosed with ICP. Optimizing maternal nutritional reserves represents a modifiable target for improving fetal growth and mitigating perinatal morbidity.

### Limitations

Several limitations should be acknowledged. The case-control design precludes causal inference. Thus, associations between dietary intake, GWG, and perinatal outcomes should be interpreted cautiously, as directionality cannot be established, and nutritional deficiencies may represent both causes and consequences of ICP. Dietary intake was self-reported using validated 24-h dietary recalls and food frequency questionnaires, which are subject to recall and reporting bias, although assessments were conducted by trained dietitians using standardized protocols. The cross-sectional assessment of nutritional status limits evaluation of temporal relationships across gestation, necessitating longitudinal studies with serial assessments. Finally, the single-center setting may limit generalizability, and the high prevalence of coexisting gestational diabetes mellitus represents a relevant confounder that should be addressed in future studies.

## CONCLUSION

ICP is associated with maternal nutritional compromise characterized by significantly reduced energy and micronutrient intake, and insufficient GWG. These nutritional inadequacies are significantly associated with adverse perinatal outcomes, including earlier gestational age at delivery, lower birth weight, and increased neonatal morbidity requiring neonatal intensive care. The positive association between maternal GWG and neonatal birth weight suggests that optimizing maternal nutritional status may provide a valuable strategy to mitigate the neonatal consequences of ICP. Integration of nutritional assessment and individualized dietary counselling into the medical management of ICP is recommended.

## Data Availability

The datasets generated and/or analyzed during the current study are available from the corresponding author upon reasonable request.
